# Uncertainties in Predicting Species Distributions under Climate Change: A Case Study Using *Tetranychus evansi* (Acari: Tetranychidae), a Widespread Agricultural Pest

**DOI:** 10.1371/journal.pone.0066445

**Published:** 2013-06-17

**Authors:** Christine N. Meynard, Alain Migeon, Maria Navajas

**Affiliations:** INRA, UMR CBGP (INRA/IRD/Cirad/Montpellier SupAgro), Campus international de Baillarguet, Montferrier-sur-Lez, France; Ghent University, Belgium

## Abstract

Many species are shifting their distributions due to climate change and to increasing international trade that allows dispersal of individuals across the globe. In the case of agricultural pests, such range shifts may heavily impact agriculture. Species distribution modelling may help to predict potential changes in pest distributions. However, these modelling strategies are subject to large uncertainties coming from different sources. Here we used the case of the tomato red spider mite (*Tetranychus evansi*), an invasive pest that affects some of the most important agricultural crops worldwide, to show how uncertainty may affect forecasts of the potential range of the species. We explored three aspects of uncertainty: (1) species prevalence; (2) modelling method; and (3) variability in environmental responses between mites belonging to two invasive clades of *T. evansi*. Consensus techniques were used to forecast the potential range of the species under current and two different climate change scenarios for 2080, and variance between model projections were mapped to identify regions of high uncertainty. We revealed large predictive variations linked to all factors, although prevalence had a greater influence than the statistical model once the best modelling strategies were selected. The major areas threatened under current conditions include tropical countries in South America and Africa, and temperate regions in North America, the Mediterranean basin and Australia. Under future scenarios, the threat shifts towards northern Europe and some other temperate regions in the Americas, whereas tropical regions in Africa present a reduced risk. Analysis of niche overlap suggests that the current differential distribution of mites of the two clades of *T. evansi* can be partially attributed to environmental niche differentiation. Overall this study shows how consensus strategies and analysis of niche overlap can be used jointly to draw conclusions on invasive threat considering different sources of uncertainty in species distribution modelling.

## Introduction

Species may respond to environmental change by either shifting their geographic range, getting extinct, or else adapting locally to the new environmental conditions through changes in phenology and behaviour [1], [2], [3]. Adding to this diversity of potential responses, range shifts, new introductions and extinctions of agricultural pests and their predators may have major consequences for food production and food security [4], [5]. This is especially true in countries where the infrastructure and governance may not be optimized to respond rapidly to pest outbreaks and yield changes [6], [7], [8]. Moreover, changes in agricultural productivity in some tropical areas may be coupled with rapid human population growth, and these changes may have synergistic negative impacts on human populations [7]. Therefore, the question of how these changes will affect agriculture throughout the world will have important economic and social consequences under climate change scenarios.

Species distribution modelling (SDM) has been used extensively to predict current species distributions and future range shifts [9], [10]. Identifying areas and ecosystems at high risk of pest outbreaks may help to redirect new efforts to control further pest spread and serve as an indicator of how vulnerable different areas may become when climatic conditions will change [11]. However, studies using SDMs rarely quantify or map uncertainty, although this has been recognized as one of the major needs in the field [12], [13], [14]. Ensemble forecasting has been advocated as a way to remediate this issue by considering several statistical modelling strategies at the same time and averaging their predictions according to the predictive ability of the models [15], [16]. Another common issue in modelling species distributions is the lack of a meaningful measure of presence-absence discrimination when presence-only data is available for modelling [17], [18], making the task of weighting different models in an ensemble forecasting approach difficult. Although some options have been proposed to measure model predictive ability when presence-only data are available [19], [20] they rarely explore the effects of varying species prevalence (i.e. the overall frequency of the species in the modelling area) in the results when a priori information regarding potential prevalence is absent, a factor that has been widely recognized to affect model predictions [21], [22], [23], [24].

The tomato red spider mite *Tetranychus evansi* is a small arthropod, first recorded in Brazil in 1952 [25] under the name of *Tetranychus marianae* and described in 1960 from Mauritius [26]. It was only after it caused significant agricultural damage in tomato cultures in the early 1980 s in Africa and Brazil that the species started being considered as a significant agricultural pest [27]. Outbreaks were said to cause up to 90% loss in tomato cultures in South-East and West Africa [28], [29]. Outbreaks were then recorded in Europe, especially around the Mediterranean basin where it has spread significantly in the last decades. The mite was later reported in Asia, mainly Japan and China (see [30] for a complete list of reports). The species is inconspicuous, with a rapid growth cycle (10 days under optimal conditions) and high tolerance to hot and dry conditions, making it a challenging pest in many agricultural systems. The species has also been misidentified in several occasions (e.g. [31]), causing a slow response to its outbreaks in newly invaded areas, an issue often reported for spider mites (e.g. [32]). Detailed recent genetic studies of different populations of *T. evansi* around the world suggest that the species is native to South America, and show that two main distinct clades, both coming from Brazil, and genetically characterized as clade 1 and 2, explain current patterns of species genetic diversity [33], [34]. The most likely scenario for the species spread involves an initial introduction of clade 1 from Brazil into Africa, and invasion of this population into the Mediterranean, with later reports in Asia. A second introduction of clade 2 occurred into a restricted area of the Mediterranean basin, with a subsequent mixture of both clades [33]. Therefore the two clades have invaded distinct geographic areas worldwide. The question that remains to be answered is whether or not this current differential distribution is solely the result of introduction events, or whether it reflects large-scale environmental niche differentiation between clades. These two scenarios have important consequences for climate change analysis, since in the absence of niche differentiation mites of the two clades of *T. evansi* would have the same invasive potential when introduced into a new area. This would be an important issue to take into account when designing strategies to prevent future pest expansion or when proposing quarantine policies.

The current study had two main objectives. The first one was to generate robust predictions of the potential distribution of the species by studying the effects of the following sources of uncertainties in model predictions: (1) the type of statistical modelling used; (2) the lack of knowledge regarding potential prevalence of the species at the global scale; and (3) the variability in environmental preferences between mites belonging to the two major invasive clades of *T. evansi* described above. The second objective was to use this knowledge to forecast the potential effects of climate change on *T. evansi*’s potential distribution using two contrasting climate change scenarios for 2080.

## Materials and Methods

### Occurrence and Environmental Datasets

Occurrence data on *T. evansi* was compiled from literature sources, mainly by screening the bibliography associated with this species in the Spider Mites Web database [35] and by adding recent observations. Part of this effort was already accomplished for previous publications [30], [36] but was here thoroughly reviewed, updated and completed. Only identifiable point locations were georeferenced, either through direct transcription of the geographic coordinates when available in the published source, or by using online geographic gazetteers and GoogleEarth to locate precisely the sampling site. Vague references to large regions, such as “present in southern Florida” were therefore discarded from this georeferencing effort. Some of the individuals sampled (mainly those coming from our own sampling efforts) were genotyped for previous studies [33], [34], revealing the existence of two clades that have invaded different geographic areas. We therefore assigned the georeferenced location points for *T. evansi* as belonging to one or the other clade when either the georeferenced individuals were the genotyped ones or when there was no ambiguity regarding their potential genotype according to their origin. This allowed us to model the species globally, as well as each clade separately.

The original occurrence data therefore included 556 point occurrences for *T. evansi*, and 418 and 107 point occurrences for the first and second clade respectively. These data were then overlaid with a 10-minute global grid, which corresponds to the resolution of the environmental data (see below). This means that a grid cell was recorded as a presence, regardless of whether one or several point occurrence records were within its limits. The resolution is also coarse enough (equivalent to 18.6 km at the equator) to reduce the importance of any geographic location error at finer scales. This left 380, 288 and 73 presence records for the species and for each clade in the distribution modelling, respectively (see Figure S1 for a map of record distributions).

Altitude as well 19 bioclimatic variables were downloaded from Worldclim at a resolution of 10 minutes for current conditions, as well as for two climate change scenarios for the year 2080 (A1B and B2A from the Canadian Centre for Climate Modelling and Analysis-CCCMA, which are based on the CGCM circulation model). These variables include mean, maximum, minimum and variability measures on temperature and precipitation [37]. The A1B scenario assumes rapid economic growth and a population that peaks in 2050, with some technologies being introduced to reduce the effects of fossil fuel (so it is a moderate scenario of climate change under high economic growth). The B2A scenario is more conservative, assuming lower population and economic growth, as well as introduction of environmental technologies, therefore producing less dramatic climatic changes projections.

Here we will explore three sources of uncertainty in model projections. The first one is the species prevalence assumed when using background data (i.e. a random sample of the available environment), rather than real absence records, and the second one is the nature of the SDM used. Therefore, in the first part we will focus on using only one type of SDM (Generalized Additive Modelling, GAM) but several prevalence levels (from 10% to 90%) whereas in the second part we will use only one species prevalence (50%) and a diversity of SDMs. The third source of uncertainty involves differential response between clades to environmental gradients, and will therefore involve analysis of niche overlap between the two clades of *T. evansi*, and projection of potential range for mites of each clade. We describe details below.

### Species Distribution Modelling

When absence data is not available, it is usually recommended that a large random sample of the available environment, called background data, is used in order to fit the models and assuming equal weights between background data and presence records [38], [39]. However, species prevalence has been shown to greatly influence model outputs [22], [24], [40], [41] so that the assumption of equal weights, equivalent to assuming 50% prevalence, should affect model predictions. We tested here what would be the effects of varying prevalence of the input data in model predictions by considering the following prevalence levels: 10, 30, 50, 70 and 90%. For all modelling efforts the presence records were complemented here with 100,000 background data randomly sampled globally so as to represent the available environment. When the modelling technique allowed it (i.e. for all methods requiring presence and absence information), presences and absences were weighted to correspond to the assumed species prevalence (e.g. for a prevalence of 50%, presences and absences weight the same, whereas for a prevalence of 90% a presence weights 9 times more than an absence). This allows including a large number of background points in order to represent the available environmental variability, but at the same time controlling for the assumed species prevalence. Notice that if the weights were not employed, we would be assuming unrealistically low species prevalence (380 presences/100,000 sites ≈ 0.004).

In all our modelling efforts, we took a General Additive Modelling approach (GAM) as our starting point. GAM allows for non-linear responses to environmental gradients, yet the level of complexity can be controlled for to avoid model over-fitting [42]. Here the maximum model complexity was fixed to quadratic relationships (k  = 3) using the *mgcv* package within R [43]. This has been shown to perform among the best modelling strategies when compared to other SDMs [44], [45]. In order to select a subset of environmental variables and avoid multi-collinearity we used a forward variable selection strategy. We therefore started by fitting a GAM model with one predictor at a time. The predictor that showed the highest explained deviance was retained in the next step, when a second predictor was added in the same manner. Before any new addition, we eliminated from the next set of potential predictors those that showed a high correlation (Pearson r ≥0.8) with the predictors already included in the model. This process of addition continued until the newly added predictor was not significant in the model or until the addition of a new variable did not produce a decrease in the Akaike Information Criterion (AIC) [46]. The variables selected in this manner were then systematically used for all other SDMs. This allowed making comparisons between model outputs knowing that all models included the same set of predictors.

To understand how much uncertainty is added in the modelling effort by species prevalence in the input data, we only used GAM, with the variables selected as explained above, and varied input prevalence on the weights using 10, 30, 50, 70 and 90% prevalence. To understand how much uncertainty is added by using different modelling techniques we kept input prevalence at 50% and used 8 different modelling strategies. Once the variables were selected using the initial GAM forward selection strategy described above, 7 other SDMs were implemented for *T. evansi* in the same dataset and using the same predictors: GLM, CART, BRT, mahalonis distance (MAHAL), BIOCLIM, DOMAIN, and MAXENT. A general description of all these methods can be found in [44] and [47]. Tree based methods (CART, BRT) and environmental distance-based methods (MAHAL, BIOCLIM, DOMAIN) were fitted using only linear terms in the predictors, whereas the regression based method (GLM) was fitted using linear and quadratic terms for all predictors in order to account for potential non-linear responses to environmental gradients. MAXENT was run with default values, and the logistic output was selected for further analysis. MAXENT, MAHAL, BIOCLIM and DOMAIN are presence-only modelling strategies [44], [48]. Therefore, they do not allow directly including the weight of background data on the output. For all other models, we used the 50% weight for presences and absences (i.e. 50% prevalence). Results from MAHAL are usually expressed as 1-distance from the environmental space where the species occur. Therefore a value of 1 represents the maximum value (i.e. no distance, meaning sites that are environmentally identical to occurrence sites) and predictions include some very large negative values. Since these values were not comparable to the ones in the model outputs for the other models (which result in a range between 0 and 1), we rescaled by linear transformation the output values from MAHAL so that the minimum corresponds to 0 and the maximum corresponds to 1 before performing any consensus or variance analysis.

### Consensus and Variance between Modelling Efforts

In the previous analyses, the outputs of each modelling exercise were summarized to look at geographic areas that represent consensus and divergence between models. Three consensus methods were initially tested. First, the predicted probability of occurrence (or suitability in the case of presence-only models) for each model (i.e. for each prevalence level and for each modelling strategy) were summarized in a principal component analysis (PCA). The first axis of the PCA represents the axis of agreement between models [49] and was used as a measure of consensus. The other two consensus maps represented the mean and median predicted probability values [50]. However, since the three methods provided very similar results (Figure S2), from now on we only focus on the median between model predictions.

Variance between model predictions was also calculated in order to represent the areas of major uncertainties, and to compare uncertainty due to prevalence versus statistical model used.

To eliminate the influence of potentially poor models, we tested model classification performance using three indices: AUC or the area under the receiver operator curve, which is an overall measure of presence and absence performance that is independent of any threshold; sensitivity (i.e. success rate at predicting presences) and specificity (i.e. success rate at predicting absences), which depend on a threshold value on predicted probabilities that is used to transform model outputs into presence-absence predictions. We used the threshold that maximizes the sum of specificity and sensitivity [51]. To perform predictions we only kept models with AUC values ≥0.9. All these measures of performance were calculated by fitting the models with 80% of the presence records and a random sample of pseudo-absences of same size, and testing the models with the other 20% of presence records and another pseudo-absence random sample of same size, all of this repeated 10 times. This reduces the inflation of the AUC and other classification success indices which, calculated with a large sample of pseudo-absences assumed to be real absences, would always appear as over-optimistic [18].

### Environmental Niche Overlap between Clades

The goal of this analysis effort was to look at whether or not the two genotypes are occupying the same environmental space. We started by carrying out a separate modelling effort for each clade by using GAM and the forward selection method described above. However, this resulted in the selection of the same environmental variables used for *T. evansi* at the species level. We therefore used the same variables, which also allowed us to compare each genotype’s occupancy along the same environmental axes. Then we looked at current potential distributions of the species and clades as well as the overlap on each environmental axis. To look at niche overlap, we used an Outlying Mean Index analysis (OMI) [52]. This is a multivariate analysis that allows studying patterns of occupancy along environmental gradients. The distribution of occurrence can have linear as well as unimodal relationships with environmental gradients [52], [53]. In a first step, an ordination technique is applied to look for combinations of environmental variables that optimize the separation between species occurrences. Niche position (mean environmental conditions of occurrence) and niche breadth (variance around the niche position) of clades along each of the ordination axes can then be calculated and compared to a random expectation by comparing the observed patterns of niche position and breadth to what would be expected from a random sample of same size from the global environment [52], [53], [54]. This provides information regarding whether or not the tested clades are specialized in some portions of the environmental gradients. In order to test whether or not there are significant differences between the two clades, we performed a pairwise t-test of occupancy along the main OMI axis, as well as along the individual environmental gradients.

## Results

### Different Prevalence versus Different Modelling Strategies

Models across prevalence levels and across modelling techniques produced very good classification success rates (Table 1 and 2), with AUC and sensitivity values generally >0.9, and specificity values >0.8.

**Table 1:** Model performance for GAM models considering different prevalence levels.

Prevalence	AUC	Sensitivity	Specificity
10%	0.947±0.019	0.922±0.029	0.889±0.041
30%	0.944±0.017	0.926±0.037	0.872±0.041
50%	0.945±0.021	0.946±0.036	0.864±0.044
70%	0.943±0.019	0.930±0.027	0.875±0.044
90%	0.918±0.025	0.912±0.043	0.826±0.057
Median	0.944±0.003	0.953±0.011	0.843±0.001

Performance is shown in terms of classification rate (AUC, sensitivity and specificity). Sensitivity and specificity correspond to those calculated using the MST threshold; the validation set used here represents 20% of the data (the other 80% was used to calibrate the models).

**Table 2:** Model performance for different statistical models.

Prevalence	AUC	Sensitivity	Specificity
GAM	0.945±0.021	0.946±0.036	0.864±0.044
GLM	0.822±0.033	0.825±0.072	0.820±0.075
BRT	0.933±0.023	0.934±0.046	0.816±0.077
CART	0.900±0.031	0.892±0.040	0.862±0.044
BIOCLIM	0.869±0.034	0.834±0.077	0.787±0.056
DOMAIN	0.847±0.031	0.825±0.062	0.728±0.078
MAHAL	0.854±0.028	0.824±0.056	0.746±0.037
MAXENT	0.952±0.006	0.942±0.024	0.865±0.015
Median	0.956±0.003	0.956±0.001	0.849±0.001

When the model allows changing species prevalence (i.e. GAM, GLM, BRT, CART), a prevalence of 50% was used in model weights. Performance is shown in terms of classification rate (AUC, sensitivity and specificity). Sensitivity and specificity correspond to those calculated using the MST threshold; the validation set used here represents 20% of the data (the other 80% was used to calibrate the models).

**Figure 1:**
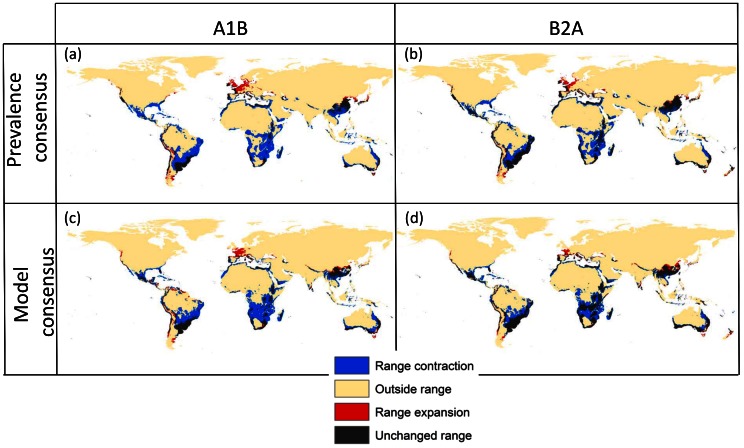
Potential range of *Tetranychus evansi*. Maps reflect median of predictions from model outputs, comparing current and future climate from two different climate change scenarios. On the left side, scenario A1B, on the right side the more conservative B2A scenario (see text for description of scenarios). The upper row shows results from consensus between GAM models with different prevalence levels (10%, 30%, 50%, 70%, 90%), whereas the lower row shows consensus between four different modelling strategies (GAM, BRT, CART, MAXENT) at a prevalence of 50%. Black areas indicate regions that are predicted as part of the potential range under current and future conditions; blue areas correspond to areas that are today part of the potential range but that are predicted to become outside the range under climate change conditions in 2080 (range contraction); red areas indicate potential range expansions meaning they become part of the potential range under climate change scenarios; finally yellow areas are not part of the potential range.

When background data was weighted to vary potential species prevalence, the resulting predictions were biased in the same way than the input data. In other words, a model built with presence weights of 10% (i.e. meaning 10% prevalence for the species globally) predicted low probability values globally in wider areas as compared to a model for which 90% prevalence was assumed (Figure S3). All model prevalence predict the core current distribution in the native range of Brazil, as well as in areas of the Mediterranean basin, Africa and Japan where the pest has already been recorded, but also areas such as southern Australia, where the species has not been recorded (Figure 1a and b).

Different modelling strategies represent quite different maps of potential distributions for current conditions (Figure S4). MAHAL and DOMAIN tend to predict much larger areas than the other modelling strategies. Since GAM, BRT, CART and MAXENT produced a higher classification success rate than the other modelling strategies (Table 2, AUC ≥0.900), we used these four models to calculate the median and predict species distributions (Figure 1c and d). This consensus projections revealed similar results than by using GAM models only with different prevalence levels (compare Figure 1a and b versus c and d). In all cases, some areas where there are no current records are included within the potential range of the species (e.g. southern Australia). Large potential range contractions were predicted in Africa and the Americas, and a range expansion into northern France and England as well as some limited areas in North America, Asia and South America were also projected under both climate change scenarios (Figure 1).

Predictions from individual GAM models with different prevalence levels, as well as the median across different prevalence levels, all predict a large contraction of the potential range of the species under both climate change scenarios (Figure 1a and b). While some areas remain part of the potential range of the species both under current and future conditions (black in Figure 1), large range contractions are predicted under climate change scenarios in Africa, North America and South America. However, range expansions into new regions, especially in northern Europe (northern France and England) are also predicted (red areas in Figure 1).

### Distribution of Uncertainty

**Figure 2:**
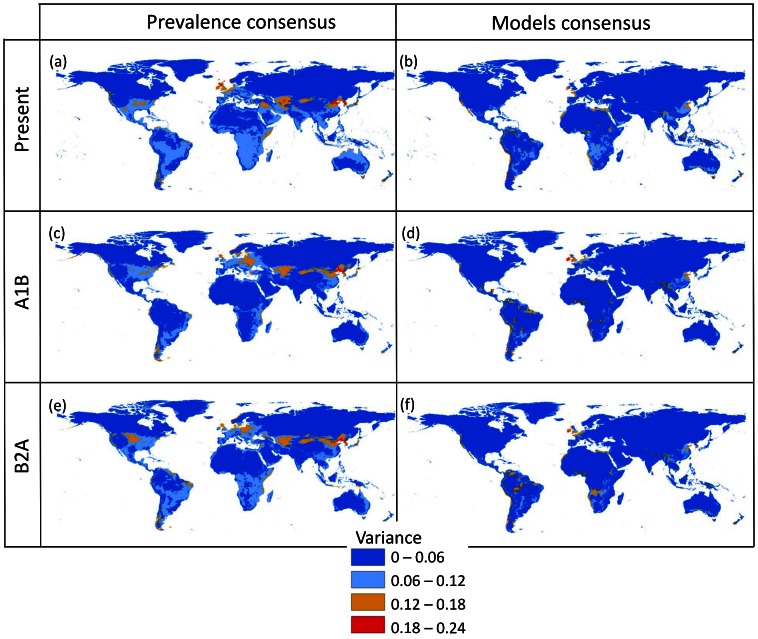
Variance between model projections computed for *T. evansi*. The left column shows variance between GAM models using varying prevalence levels (10%, 30%, 50%, 70%, 90%) whereas the right side shows variance among four different modelling strategies (GAM, BRT, CART, MAXENT) at a prevalence of 50%. Red areas indicate higher uncertainty with respect to blue areas where different models produce similar results.

Both under current and future climatic conditions, the use of different statistical models produced lower variance among predictions than varying prevalence levels when the median of the four best models were used (Figure 2), but the opposite trend was observed when all model strategies were considered (Figure S5). Prevalence produces areas of high uncertainty in Eastern China and Korea, in Central Asia, and northern England, as well as some other limited regions in eastern Africa, Europe and in the Americas (Figure 2a). Modelling strategy produces smaller areas of high variance in roughly the same places (Figure 2b). This pattern remains valid across both scenarios of climate change, with prevalence always producing more variance between predictions than using different modelling strategies when the best models are used in the calculation of consensus (Figure 2, first versus second columns).

### Environmental Niche Differentiation between Clades

**Figure 3:**
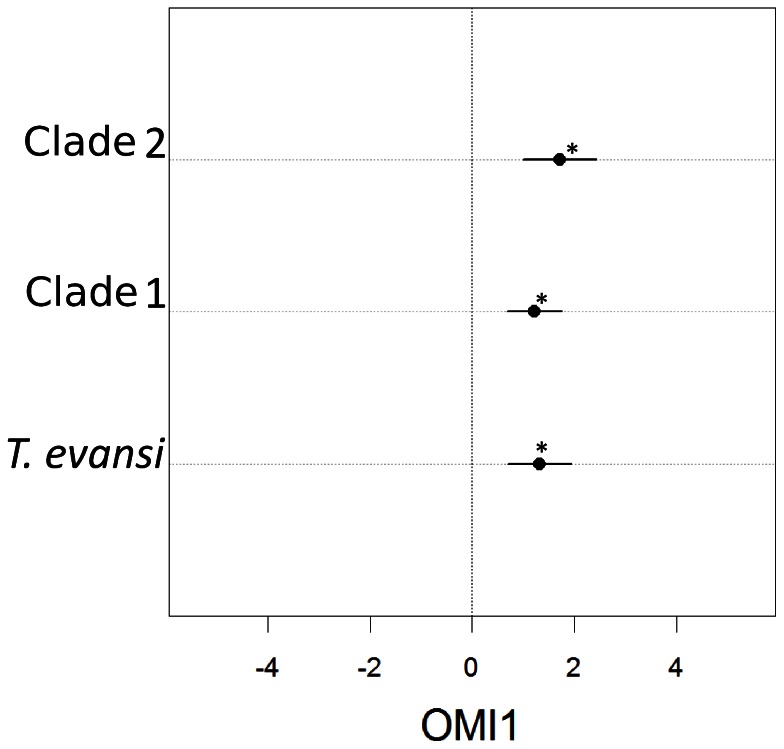
Results from the Outlying Mean Index (OMI) analysis along the first axis of ordination. The vertical line indicates the mean of 100,000 sites sampled randomly from the globe. The species as well as the two clades have their mean (black dots) as well as the standard deviation (lines around the circle) in positive values for OMI 1, showing that the species occupies warm and highly variable environments. This specialization is significant for all groups (bootstrap with respect to random samples of globally available environments, one-tailed test, *p-value* <0.001), as indicated by *. A pairwise comparison of sites occupied by clade 1 and clade 2 also shows that clade 1 occupies significantly lower values along OMI 1 axis.

**Figure 4:**
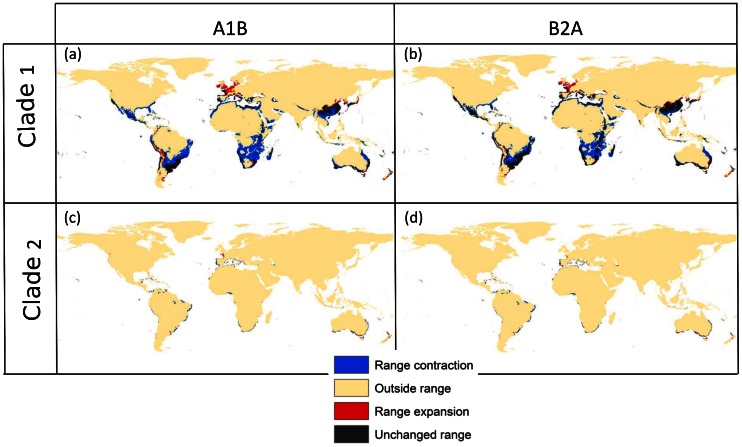
Potential range of mites of the two clades of *T. evansi* under current and future conditions. Maps reflect predictions using a GAM modelling and assuming 50% prevalence. Black areas indicate regions that are predicted as part of the potential range both under current and future conditions; blue areas correspond to areas that are today part of the potential range but that are predicted to become outside the range under climate change conditions in 2080 (range contraction); red areas indicate potential range expansions meaning they become part of the potential range under climate change scenarios; finally yellow areas are not part of the potential range.

The first axis in the OMI analysis explained a large percentage of the variation (96%) on environmental occupancy patterns between clades. Positive values along OMI 1 represented environments with low temperature range (annual and diurnal), low altitude, high annual temperature and precipitation, and low precipitation during the coldest quarter among others, temperature range and mean annual temperature being dominant in this relationship (Table 3). *Tetranychus evansi* as a whole, as well as each clade, occupied a limited range of values on the positive side of this gradient but far from the extreme available OMI 1 values, with clade 2 having a larger OMI 1 mean with respect to clade 1 (Figure 3), i.e. clade 2 occupied warmer environments with lower temperature annual ranges (see also Table S1). A bootstrap of randomly drawn available environmental conditions, compared to observed occupied sites confirms that the species and the two clades are significantly specialized along this axis (one tailed bootstrap test, *p-value* <0.001). A pairwise comparison between the two clades also showed that their occupancy along the first OMI axis was significantly different (one-sided t-test, *p-value* <0.001), clade 1 specializing in sites representing lower values of OMI 1 as compared to clade 2 (Figure 3). When the environmental variables composing OMI 1 were tested individually, all showed significant differences between the clades (one-sided t-test, *p-value* <0.05) except mean annual precipitation and precipitation during the driest quarter. These differences in environmental space are translated into dramatically different potential ranges of the two clades globally (Figure 4), with mites of clade 1 having a much larger potential range than those of clade 2, both under current and future climate change scenarios.

**Table 3:** Loadings for the first Outlying Mean Index (OMI).

Variable name	OMI 1
Temperature Annual Range	−0.976
Mean Altitude	−0.194
Mean Temperature Diurnal Range	−0.134
Precipitation of Driest Quarter	0.071
Precipitation Seasonality	0.160
Precipitation of Coldest Quarter	0.177
Annual Precipitation	0.322
Annual Mean Temperature	0.767

The first OMI axis explains most of the variation in occupancy patterns between the two clades of *T. evansi*. Loadings for the different environmental predictors are shown in this table.

## Discussion

We showed here that although modelling strategy and species prevalence have major effects on the species distribution forecasts, consensus strategies allow drawing robust conclusions regarding core distribution areas and uncertainty can be mapped. Niche overlap analysis shows that invasive mites of both clades of *T. evansi* specialize in warm environments, but that those of clade 1 have a much larger potential range than the others. Both clades would therefore present very different invasive potentials when introduced into new areas. Overall, this study provides an applied example of how the joint use of species distribution modelling and niche overlap analysis can be used to study current and future invasive potential of species considering different sources of uncertainty.

Our study reveals several important aspects of the species distribution modelling process and its use in invasive risk assessments for a significant agricultural pest under climate change scenarios. First, once the best modelling strategies are chosen with respect to their predictive ability (i.e. classification rate success), model uncertainty due to lack of knowledge on potential prevalence of the species has a larger impact than modelling strategy (Figure 2). The idea that modelling strategy has a large influence on model predictions is not new. Several recent studies have shown that it is important to incorporate such uncertainties, especially under scenarios of climate change [12], [55], [56]. For example, García *et al.* [57] studied consensus among seven modelling strategies for 2,500 animal species across Africa and using three climate models and three emission scenarios. Their results showed that the uncertainty associated to the modelling strategy was higher than that associated to the different climate change scenarios. However, the effects of assuming 50% prevalence in their input data were not evaluated. Here we showed that selecting fewer modelling strategies according to their classification rate success is a powerful means of reducing model uncertainties. However, the uncertainty due to the assumed species prevalence levels in the input data may have important consequences for model predictions. Species prevalence has long been known to influence model performance and model outputs [58], [59]. However, current recommendations regarding how to handle presence-only data usually ignore this aspect of the modelling process and recommend using 50% prevalence (e.g. [38], [57]) or the use of a correction formula on model outputs that is also ultimately influenced by the assumptions linked to species prevalence [21], [23], [24]. Here we have taken a pragmatic approach, directly varying input species prevalence in order to assess its impact on model predictions. As expected, model outputs were biased in the same direction than model inputs: models fitted with lower species prevalence produced smaller areas of high suitability than those that were fitted using higher species prevalence in the input data (Figure S3). We would therefore recommend implementing the same type of ensemble forecasting approach that has been recommended to summarize consensus among modelling strategies (e.g. [16]), but adding species prevalence as a second factor. A simple pragmatic approach would involve first implementing a classic ensemble forecasting using 50% prevalence in order to select the best modelling strategies, and then varying species prevalence using the best modelling approach. This would allow considering differences among modelling strategies as well as effects of species prevalence on model forecasting. Expert opinion may reveal to be very useful in limiting the initial choices in this procedure, especially when species prevalence may have a large influence on the geographic extent of the potential distribution of the species and when there is some biological knowledge regarding factors limiting the species survival in the field.

Second, uncertainty is not distributed homogeneously across space: areas of large uncertainty can be mapped and identified, and are much reduced with respect to areas of certainty (Figure 2). These areas may be related to regions where future climate shows different properties than the current climate. For example Zurell *et al.* [60] showed that large differences among models were generated when the range of the variables used in the fitting process was smaller than the range used in the projection phase. In this case BRT will generate a flat response outside the fitting range, while a GAM will extrapolate the shape of the relationship as a continuation of the range of values used to fit the model. As shown in that study, this generates situations where new conditions also generate higher uncertainty in model projections. In our case, a large background sample was used to fit the models, so that current conditions are fairly well represented. This risk is therefore likely to influence uncertainty under future scenarios more than under current conditions. However, high areas of uncertainty under current and future conditions largely overlap (Figure 2), suggesting that other factors are also at play. The influence of prevalence is especially notorious at the edge of the potential range (left side in Figure 2): if we were to assume higher prevalence levels, the edge towards more temperate regions would be more likely to change from unsuitable to suitable, whereas large areas in the tropics and at the poles remain largely consistent across all model prevalence. This means that the direction and extent of this uncertainty could be potentially predictable and would allow adjusting the models as new invasions unfold, and also focusing monitoring efforts in these regions in order to determine the species real potential.

Third, although both climate change scenarios used here are quite different in terms of their economic and environmental assumptions, they both produced very similar predictions in terms of the potential distribution of *T. evansi* (Figure 1 and 4). This result echoes that of García *et al*. [57], suggesting that modelling strategy has a larger influence than climate change scenarios at this scale. In this situation, a simple average between conditions may be sufficient to account for climate model uncertainties. As in the case of the effects of prevalence, identifying the few areas where both climate change scenarios produce different predictions would also help set up targeted monitoring efforts that would help refine the models and generate more concrete management recommendations for particular pests.

Finally, the analyses of niche overlap among the two main clades of *T. evansi* that have invaded different regions of the world suggest that both mites occupy indeed environments with different characteristics. Although the patterns of genetic diversity demonstrate that current distributions of these clades can be explained by the history of introductions [33] the fact that they both occupy environments with different characteristics suggests that niche differentiation has also played a role in the current range of the species. This suggests that the invasive potential of one of them is much greater than the other, which is supported by their distribution range in the invaded continents, with one of the clades (clade 2) having been found so far in only a very restricted area in Southern Europe [33]. The fact that it is not only mean temperature but also temperature range that influences species distribution may point to more complex physiological responses to climate change, especially because climate variability is predicted to increase in parallel to average temperature. Undergoing experimental work regarding temperature response of mites belonging to the two clades goes in the same direction, demonstrating that the most invasive clade is capable of tolerating much lower minimal temperatures (unpublished data).

Tropical species are increasingly moving around the world and biological invasions are regarded as a pervasive consequence of global change and international trade [61], [62], a trend that also applies to spider mites. The introductions of tropical species of spider mites into Europe has increased by 50% in the last 30 years [63], the most threatened region in Europe today being the Mediterranean basin where spider mites feed on subtropical and tropical crops, such as avocados and citrus [63]. Overall, the idea that *T. evansi* will decrease its potential range globally but increase as a threat to some new areas in northern Europe remains fairly well supported across models and climate change scenarios despite variability between clades. This would be beneficial for agriculture in tropical countries but troublesome in northern Europe. However, this result needs to be considered with caution. Extreme temperatures in tropical areas may decrease overall agricultural productivity in these regions through direct effects of extremely high temperatures on plants [64], [65]. However, current efforts to model the impacts of climate change on agricultural yield usually focus on the effects of climate on plant productivity, and ignore potential interactions with agricultural pests or human management responses [8], [66]. More general studies on biodiversity and climate change have also pointed to a potential decrease of ectotherm diversity in the tropics under warmer conditions [67]. This may play in favour of agriculture when pests such as the red spider mites are projected to get extinct in some areas, but the final balance will also depend on the composition of the new arthropod community, with potential extinction of competitors and predators as well. What these results mean in the wider context of food security under climate change scenarios is that a large multi-trophic modelling effort is well overdue in order to identify pests that may represent an increased risk under new environmental conditions, so that we can plan and monitor in advance. Agricultural adaptation in tropical areas will likely involve a combination of strategies, including the use of drought and heat resistant varieties of plants where arthropod pests may become less of a concern, whereas temperate areas will likely require more monitoring and control of pests spreading from tropical areas.

## Supporting Information

Figure S1 **Map showing the distribution of occurrences used in the modelling process.**
(PDF)

Figure S2 **Results from different consensus strategies (mean, median and PCA between predictions) for current climate conditions.**
(PDF)

Figure S3 **Model predictions using different prevalence levels under current climate conditions.**
(PDF)

Figure S4 **Predicted probability of occurrence for each model type under current climate conditions.**
(PDF)

Figure S5 **Variance between model predictions comparing current and future conditions, varying prevalence and modelling strategy when the models with lower classification rates are considered.**
(PDF)

Table S1 Environmental mean and standard deviation of environmental conditions in sites occupied by each of the two identified clades of *T. evansi*.
(PDF)
