# Supplementary material for: Uncertainties in Predicting Species Distributions under Climate Change: A Case Study Using Tetranychus evansi (Acari: Tetranychidae), a Widespread Agricultural Pest
Source: PLoS One. 2013 Jun 17;8(6):e66445. doi: 10.1371/journal.pone.0066445 (PMC3684581; doi:10.1371/journal.pone.0066445)

Figure S1: Map showing the distribution of occurrences used in the modelling process. Occurrence records that could not be assigned with certainty to one or the other clade are shown as "others" and were used to model the species but not the clades' potential range.

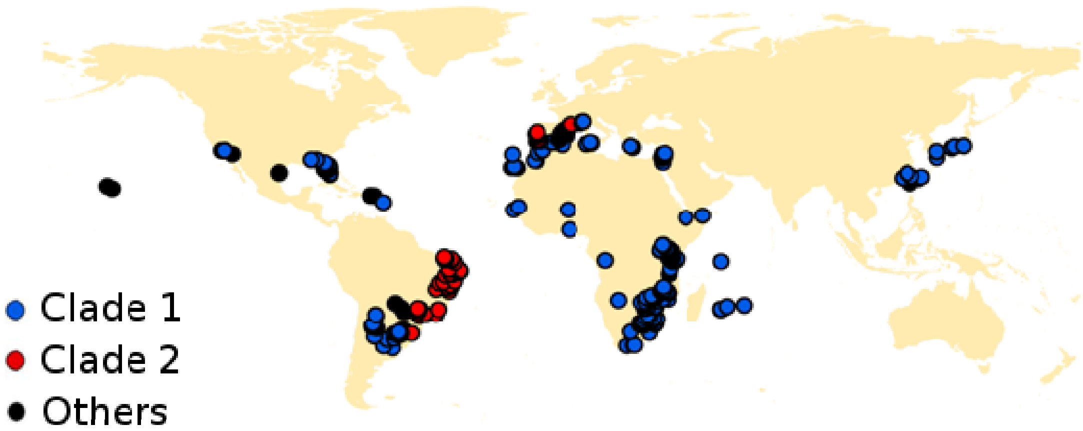

Supplement: Figure S1 — Map showing the distribution of occurrences used in the modelling process. (PDF) [file pone.0066445.s001.pdf]
