# Supplementary material for: Uncertainties in Predicting Species Distributions under Climate Change: A Case Study Using Tetranychus evansi (Acari: Tetranychidae), a Widespread Agricultural Pest
Source: PLoS One. 2013 Jun 17;8(6):e66445. doi: 10.1371/journal.pone.0066445 (PMC3684581; doi:10.1371/journal.pone.0066445)

Figure S2: Results from different consensus strategies (mean, median and PCA between predictions) for current climate conditions. The three maps are fairly similar, and therefore we focused on median in the article.

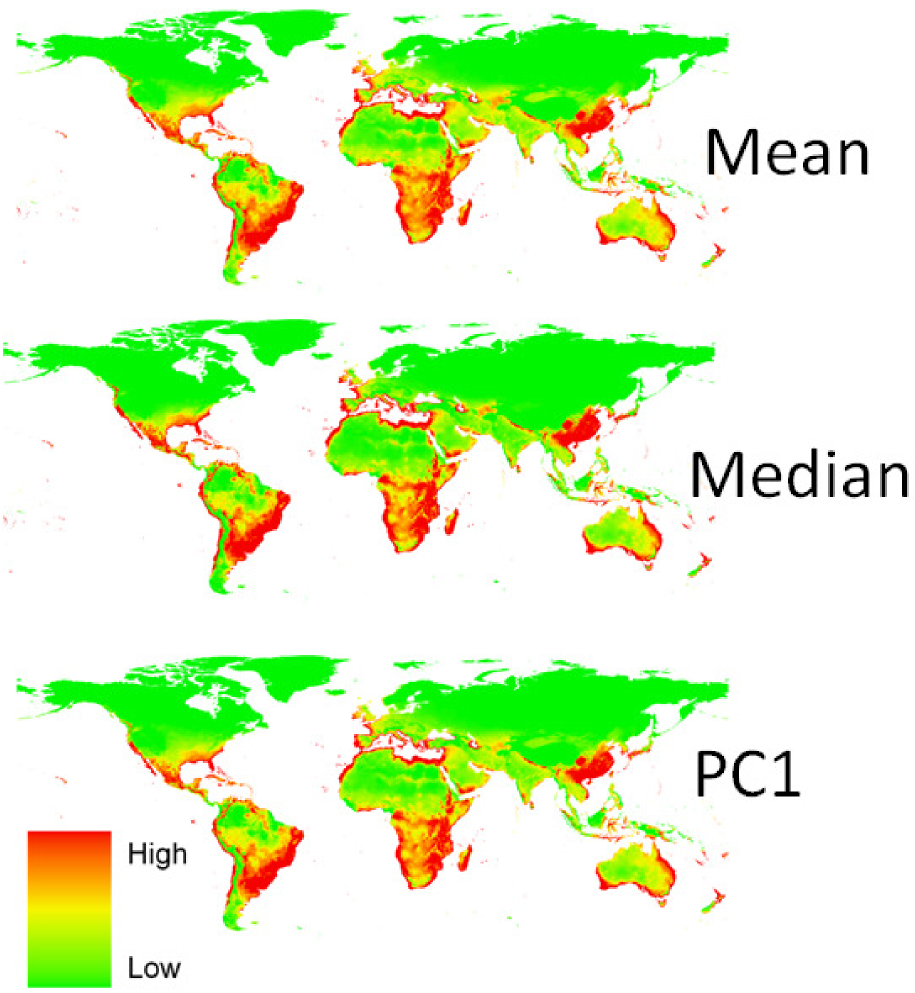

Supplement: Figure S2 — Results from different consensus strategies (mean, median and PCA between predictions) for current climate conditions. (PDF) [file pone.0066445.s002.pdf]
