# Supplementary material for: Uncertainties in Predicting Species Distributions under Climate Change: A Case Study Using Tetranychus evansi (Acari: Tetranychidae), a Widespread Agricultural Pest
Source: PLoS One. 2013 Jun 17;8(6):e66445. doi: 10.1371/journal.pone.0066445 (PMC3684581; doi:10.1371/journal.pone.0066445)

Figure S3: Model predictions using different prevalence levels under current climate conditions. Notice that the model outputs are biased in the same direction than the input prevalence, with a prevalence of 10% producing a smaller potential range than higher prevalence levels.

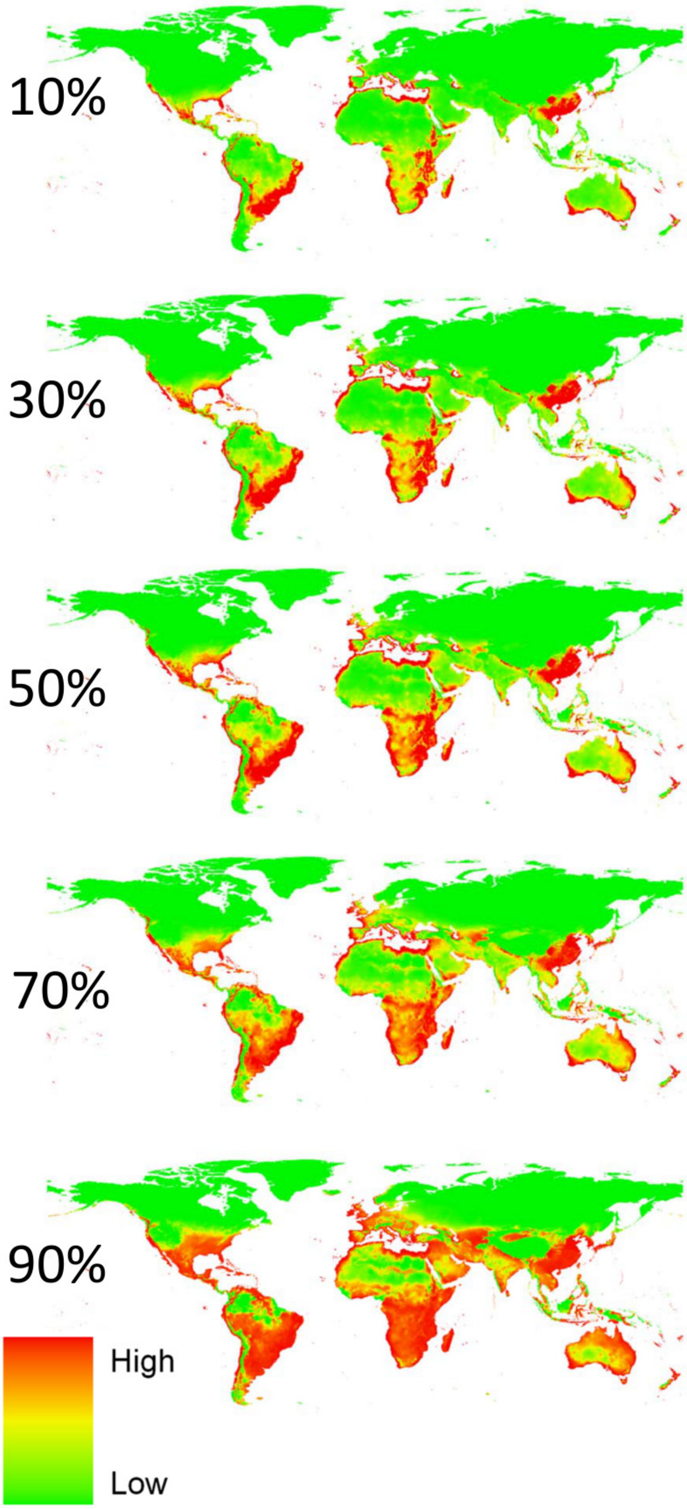

Supplement: Figure S3 — Model predictions using different prevalence levels under current climate conditions. (PDF) [file pone.0066445.s003.pdf]
