# Supplementary material for: Uncertainties in Predicting Species Distributions under Climate Change: A Case Study Using Tetranychus evansi (Acari: Tetranychidae), a Widespread Agricultural Pest
Source: PLoS One. 2013 Jun 17;8(6):e66445. doi: 10.1371/journal.pone.0066445 (PMC3684581; doi:10.1371/journal.pone.0066445)

Figure S4: Predicted probability of occurrence for each model type under current climate conditions. Notice that DOMAIN and MAHAL produce much higher probabilities in large areas where the other models are more conservative, systematically over-predicting the potential range of the species.

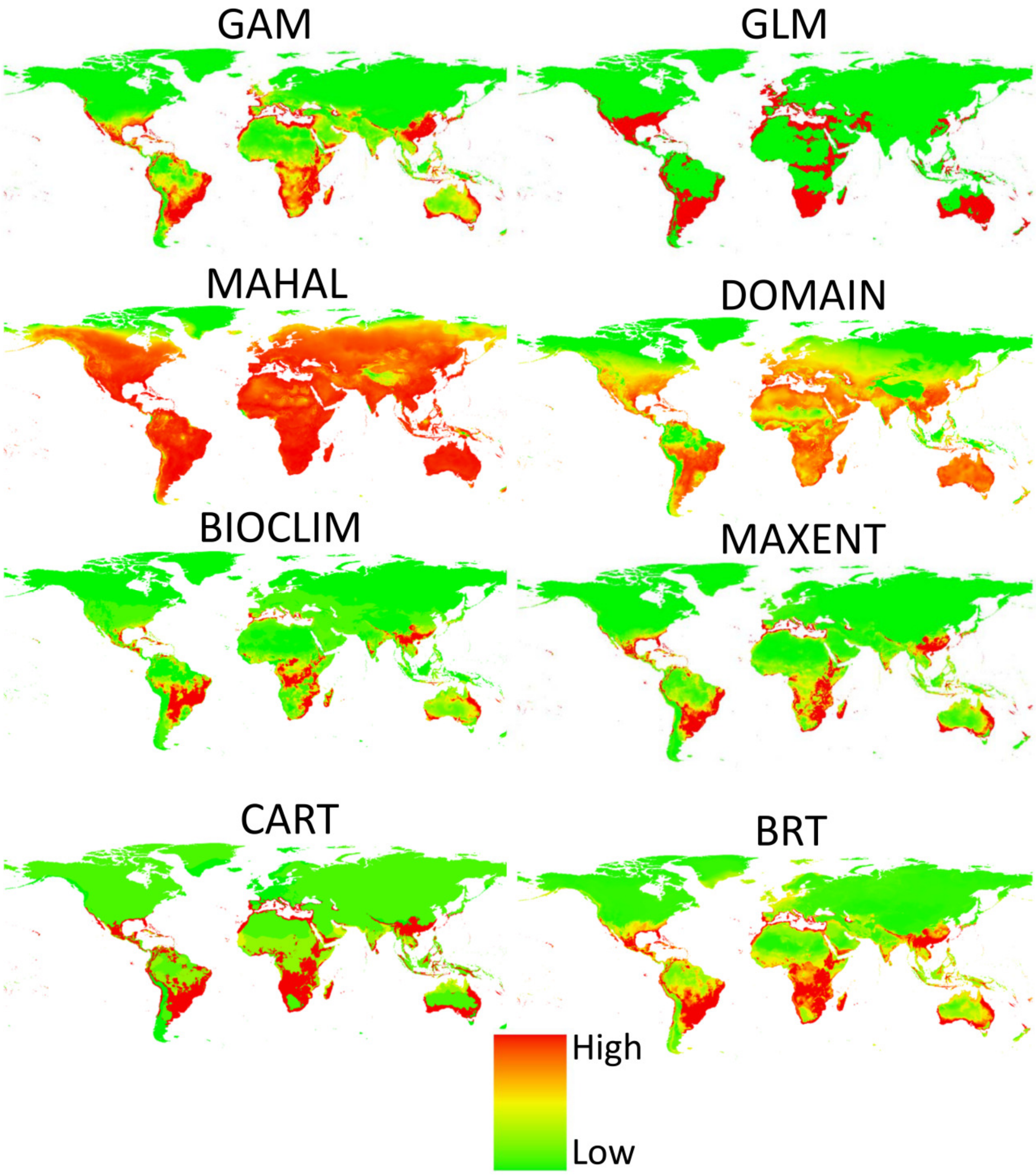

Supplement: Figure S4 — Predicted probability of occurrence for each model type under current climate conditions. (PDF) [file pone.0066445.s004.pdf]
