# Supplementary material for: Uncertainties in Predicting Species Distributions under Climate Change: A Case Study Using Tetranychus evansi (Acari: Tetranychidae), a Widespread Agricultural Pest
Source: PLoS One. 2013 Jun 17;8(6):e66445. doi: 10.1371/journal.pone.0066445 (PMC3684581; doi:10.1371/journal.pone.0066445)

Figure S5: Variance between model predictions comparing current and future conditions, varying prevalence and modelling strategy when the models with lower classification rates are considered. On the left, variance between GAM models considering different prevalence levels (10, 30, 50,70 and 90%); on the right, variance considering 50% prevalence with different modelling strategies. This figure is the equivalent to Figure 3 in the article except that here all models, regardless of their classification rate performance, are considered on the right side (whereas in Figure 3 only the consensus among the four best models is considered).

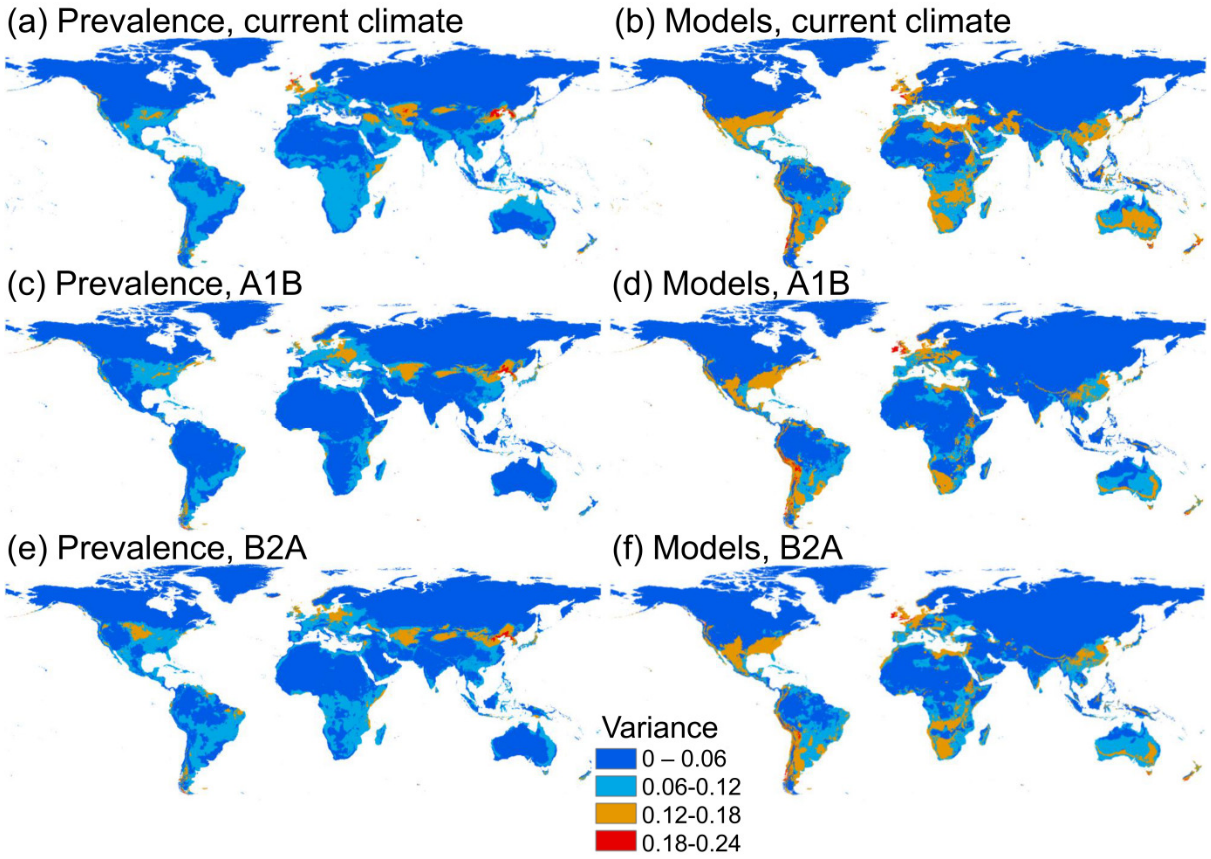

Supplement: Figure S5 — Variance between model predictions comparing current and future conditions, varying prevalence and modelling strategy when the models with lower classification rates are considered. (PDF) [file pone.0066445.s005.pdf]
