# Supplementary material for: Uncertainties in Predicting Species Distributions under Climate Change: A Case Study Using Tetranychus evansi (Acari: Tetranychidae), a Widespread Agricultural Pest
Source: PLoS One. 2013 Jun 17;8(6):e66445. doi: 10.1371/journal.pone.0066445 (PMC3684581; doi:10.1371/journal.pone.0066445)
Supplement: Table S1 — Environmental mean and standard deviation of environmental conditions in sites occupied by each of the two identified clades of T. evansi. (PDF) [file pone.0066445.s006.pdf]

Table S1: Environmental mean and standard deviation of environmental conditions in sites occupied by each clade (only variables used in the modelling process are shown). Notice that since values were extracted from Worldclim (www.worldclim.org), temperature values correspond to real temperature in Celsius x 100.

**Variable**	**Clade 1**	**Clade 2**
Mean altitude (m)	569.264 ± 594.778	223.438 ± 239.587
Annual Mean Temperature (°C x 100)	193.295 ± 30.076	205.507 ± 45.856
Mean Diurnal Temperature Range (°C x 100)	110.545 ± 28.715	92.288 ± 19.451
Temperature Annual Range (°C x 100)	219.125 ± 50.225	175.096 ± 60.795
Annual Precipitation (mm)	927.427 ± 549.630	1010.808 ± 422.098
Precipitation Seasonality	68.083 ± 28.056	59.616 ± 25.026
Precipitation of Driest Quarter (mm)	78.326 ± 91.870	91.904 ± 77.307
Precipitation of Coldest Quarter (mm)	134.830 ± 114.032	271.644 ± 209.725
